# Clinical characteristics and risk factors of cardiac surgery associated-acute kidney injury progressed to chronic kidney disease in adults: A retrospective, observational cohort study

**DOI:** 10.3389/fcvm.2023.1108538

**Published:** 2023-03-08

**Authors:** Xiaoguang Fan, Zehua Shao, Suhua Gao, Zhenzhen You, Shuai Huo, Zhu Zhang, Qiuhong Li, Saijun Zhou, Lei Yan, Fengmin Shao, Pei Yu

**Affiliations:** ^1^ NHC Key Laboratory of Hormones and Development, Chu Hsien-I Memorial Hospital and Tianjin Institute of Endocrinology, Tianjin Medical University, Tianjin, China; ^2^ Tianjin Key Laboratory of Metabolic Diseases, Tianjin Medical University, Tianjin, China; ^3^ Department of Nephrology, Henan Provincial People’s Hospital, Department of Nephrology of Central China Fuwai Hospital, Central China Fuwai Hospital of Zhengzhou University, Zhengzhou, China; ^4^ Heart Center of Henan Provincial People’s Hospital, Central China Fuwai Hospital, Central China Fuwai Hospital of Zhengzhou University, Zhengzhou, China; ^5^ Department of Nephrology, Henan Provincial People's Hospital, Henan Key Laboratory for Renal Immunity, People's Hospital of Zhengzhou University, Zhengzhou, China

**Keywords:** cardiac surgery associated-acute kidney injury, chronic kidney disease, risk factors, prediction, clinical characteristics

## Abstract

**Introduction:**

To retrospectively investigate the clinical characteristics and risk factors of cardiac surgery associated-acute kidney injury (CS-AKI) progressed to chronic kidney disease (CKD) in adults and to evaluate the performance of clinical risk factor model for predicting CS-AKI to CKD.

**Methods:**

In this retrospective, observational cohort study, we included patients who were hospitalized for CS-AKI without a prior CKD [estimated glomerular filtration rate (eGFR) < 60 ml · min^−1^·1.73 m^−2^] at Central China Fuwai Hospital from January 2018 to December 2020. Survived patients were followed up for 90 days, the endpoint was CS-AKI to CKD, and then divided them into two groups (with or without CS-AKI to CKD). The baseline data including demographics, comorbidities, renal function, and other laboratory parameters were compared between two groups. The logistic regression model was used to analyze the risk factors for CS-AKI to CKD. Finally, receiver operator characteristic (ROC) curve was drawn to evaluate the performance of the clinical risk factor model for predicting CS-AKI to CKD.

**Results:**

We included 564 patients with CS-AKI (414 males, 150 females; age: 57.55 ± 11.86 years); 108 (19.1%) patients progressed to new-onset CKD 90 days after CS-AKI. Patients with CS-AKI to CKD had a higher proportion of females, hypertension, diabetes, congestive heart failure, coronary heart disease, low baseline eGFR and hemoglobin level, higher serum creatinine level at discharge (*P *< 0.05) than those without CS-AKI to CKD. Multivariate logistic regression analysis revealed that female sex(*OR *= 3.478, 95% *CI*: 1.844–6.559, *P *= 0.000), hypertension (*OR *= 1.835, 95% *CI:* 1.046–3.220, *P *= 0.034), coronary heart disease (*OR *= 1.779, 95% *CI:* 1.015–3.118, *P *= 0.044), congestive heart failure (*OR *= 1.908, 95% *CI:* 1.124–3.239, *P *= 0.017), preoperative low baseline eGFR (*OR *= 0.956, 95% *CI:* 0.938–0.975, *P *= 0.000), and higher serum creatinine level at discharge (*OR *= 1.109, 95% *CI:* 1.014–1.024, *P *= 0.000) were independent risk factors for CS-AKI to CKD. The clinical risk prediction model including female sex, hypertension, coronary heart disease, congestive heart failure, preoperative low baseline eGFR, and higher serum creatinine level at discharge produced a moderate performance for predicting CS-AKI to CKD (area under ROC curve = 0.859, 95% *CI:* 0.823–0.896).

**Conclusion:**

Patients with CS-AKI are at high risk for new-onset CKD. Female sex, comorbidities, and eGFR can help identify patients with a high risk for CS-AKI to CKD.

## Introduction

Acute kidney injury (AKI) is considered an emerging health problem due to its high incidence, mortality, and disability rate worldwide (1). The incidence of AKI in hospitalized patients is 11.6% (2). Furthermore, AKI is associated with increased long-term poor outcomes such as chronic kidney disease (CKD) and end-stage renal disease (3–6). Cardiac surgery related-acute kidney injury (CS-AKI) is a common serious complication in patients after cardiac surgery. Moreover, AKI occurs in up to 30% of patients who undergo cardiac surgery; even a slight increase in the postoperative serum creatinine level increases hospital costs and mortality (7). A strong association exists between the severity of AKI and its later progression to CKD. Moreover, even a slight increase in the postoperative serum creatinine levels is significantly associated with an increased risk of subsequent CKD and CKD progression (8–11), which seriously affects the prognosis of patients who undergo cardiac surgery and is associated with adverse outcomes, such as prolonged hospital stay, progression to CKD, and even dialysis dependence (12). Systematic evaluation of renal function recovery 3 months or even longer after the onset of AKI helps judge the prognosis of patients and early prevention and treatment of CKD (13, 14). The current research on CS-AKI mainly focuses on the evaluation of risk factors, the establishment of prediction models, and the occurrence and short-term consequences of AKI (in-hospital death and hemodialysis). Few small-sample studies on the long-term prognosis of CS-AKI progression to CKD are available. Therefore, our study established a retrospective cohort of hospitalized patients with CS-AKI, took the progression to CKD 90 days after the occurrence of CS-AKI as the research endpoint, paid attention to the clinical characteristics and related risk factors of progression to CKD after the occurrence of CS-AKI, and established a clinical risk prediction model for CS-AKI progression to CKD. The evaluation of its predictive performance can help identify high-risk patients with CS-AKI progressing to CKD, provide early intervention for subsequent renal monitoring and treatment, and reduce the risk of the special group of patients who develop CKD after CS-AKI.

## Methods

1. Subjects and groups: In this retrospective, observational cohort study, we included patients who underwent cardiac surgery (coronary bypass, valve replacement, coronary bypass combined with valve replacement surgery, and type A aortic dissection surgery) at Central China Fuwai Hospital from January 2018 to December 2020, had CS-AKI events after surgery and without a prior CKD (eGFR < 60 ml · min^−1^·1.73 m^−2^), survived and were discharged from hospital and followed up for >90 days. Patients aged ≥18 years who met the diagnostic criteria for AKI in the 2012 AKI diagnosis and treatment guidelines of the Kidney Disease Improvement Organization (KDIGO) in 2012 (15) without a prior CKD(eGFR < 60 ml · min^−1^·1.73 m^−2^) were included. The patients with CS-AKI who survived and were discharged from the hospital were followed up for at least 90 days after discharge. Patients with a baseline eGFR of <60 ml · min^−1^ · 1.73 m^−2^; previous history of CKD; positive proteinuria; preoperative diagnosis of AKI based on CKD (A on C); urinary tract infection; and obstruction or malignant tumor and pregnant women were excluded. The patients were divided into two groups based on whether they progressed to CKD 90 days after the occurrence of CS-AKI: CS-AKI-CKD and CS-AKI-NCKD groups. The study protocol was approved by the Ethics Committee on Oct. 9, 2020 (No.136) in Henan Provincial People's Hospital. The patients provided their written informed consent to participate in this study.
2. Diagnosis and staging of AKI (1): According to the (Kidney Disease Improving Global Outcomes, KDIGO) 2012 AKI diagnosis and treatment guidelines (15) serum creatinine criteria, the diagnosis of AKI can be made based on one of the following conditions: a. Serum creatinine level rises to ≥26.5 µmol/L (0.3 mg/dl) within 48 h and b. 7-day internal serum creatinine level increased to >1.5 times the baseline level (2). KDIGO2012 AKI staging criteria (15): AKI stage 1: serum creatinine increased to 1.5–1.9 times the baseline level or increased by ≥26.5 µmol/L; AKI stage 2: serum creatinine increased to 2.0–2.9 times the baseline level; AKI stage 3: serum creatinine increased to >3 times the baseline level or ≥354 µmol/L; or renal replacement therapy was started.
3. Clinical data collection and patient follow-up: After enrolling the patients, we collected their basic information: demographic data, history of preoperative comorbidities, such as heart failure(defined as NYHA functional Class II or greater) hypertension, previous myocardial infarction, diabetes mellitus(use of oral glucose-lowering agents or insulin), cerebrovascular disease, peripheral vascular disease, chronic obstructive pulmonary disease(COPD), previous heart surgery; and medications, and physical examination findings; left ventricular ejection fraction(LVEF) level, glycosylated hemoglobin(%), serum creatinine(µmol/L); hospitalization measures; surgical methods, including coronary artery bypass grafting, valve replacement, coronary artery bypass grafting combined with valve replacement, and aortic dissection type A aortic arch replacement; operation time; intraoperative blood loss; whether to perform cardiopulmonary bypass(CPB), intra-aortic balloon pump(IABP)use, or continuous renal replacement therapy(CRRT) after the operation; blood transfusion within 14 days; occurrence of infection; occurrence of atrial fibrillation; and ventilator-assisted ventilation time. For those who survived CS-AKI and were discharged, the serum creatinine level 90 days after the occurrence of CS-AKI was followed up through regular outpatient clinics and telephone calls; eGFR was calculated based on the creatinine levels using the Chronic Kidney Disease Epidemiology Collaboration (CKD-EPI) equation (16).
4. Study endpoint: The primary endpoint of this study was the progression of CS-AKI to CKD, which was defined as eGFR < 60 ml · min^−1^ · 1.73 m^−2^ at 90 days after the onset of AKI, according to the KDIGO 2012 AKI diagnosis and treatment guidelines (15).
5. Statistical methods: Statistical Package for Social Science (SPSS) for Windows, version 20(SPSS Inc., Chicago, IL, USA) was used for statistical analysis. Measurement data were tested for normality: normally distributed data were expressed in the form of x ± s and non-normally distributed data were expressed in the form of M (1/4, 3/4). The independent samples *t*-test (normal distribution) was used for comparison between the two groups; the Mann–Whitney *U* test was used for non-normal distribution. Count data or rank data were expressed as frequency and percentage (%). The Pearson chi-square test or corrected chi-square test was used for comparison between the two groups. The clinical influencing factors of CS-AKI progression to CKD were analyzed using the univariate logistic regression analysis; the indexes with *P* < 0.05 in the results were included in the multivariate logistic regression model by stepwise method to find independent risk factors that could predict the progression of CS-AKI to CKD. The Hosmer–Lemeshow method was used to test the goodness of fit and stability of the logistic regression model, and *P* > 0.05 was regarded as an acceptable model. The area under the receiver operating characteristic curve was used to assess the predictive power of the clinical risk prediction model for progression from CS-AKI to CKD.

## Results

**Figure 1:**
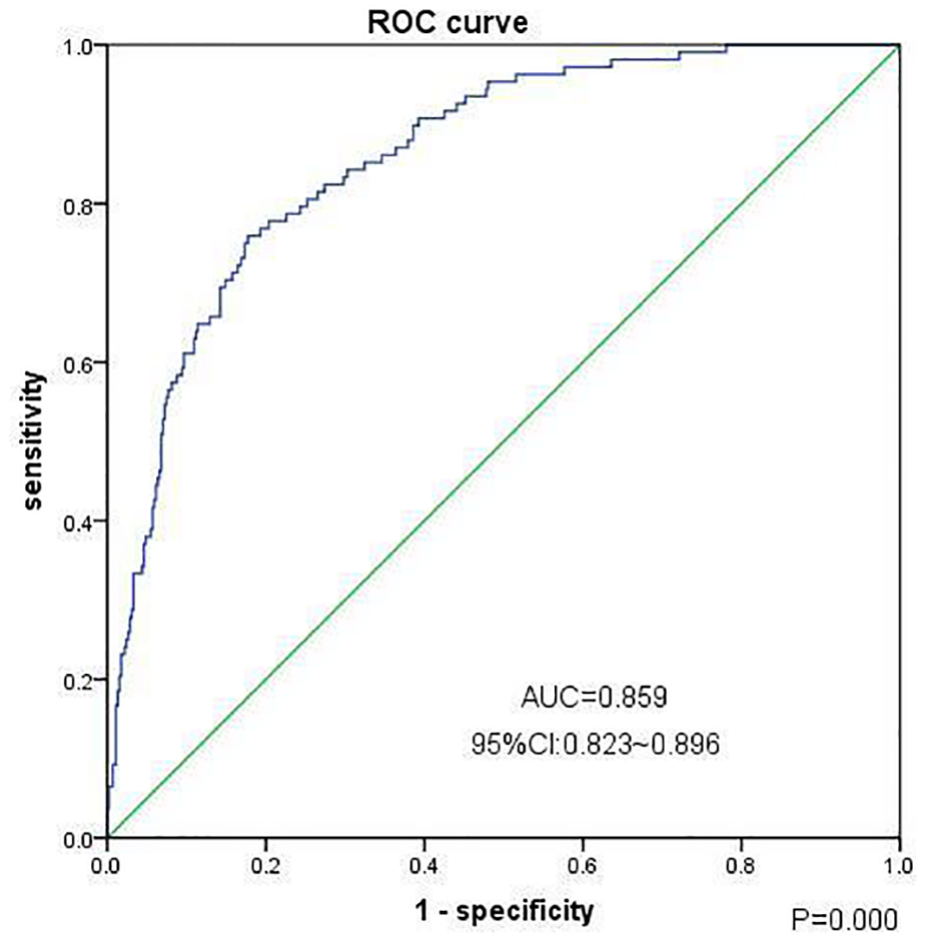
Clinical prediction model (ROC curve) for the progression of chronic kidney disease in patients 90 days after CS-AKI. Note: The Goodness of Fit Hosmer-Lemeshow test, Chi-square value 5.693, *P *= 0.682, cutoff value = 0.226, *n_p_*_ < cutoff _= 401, *n_p_*_ ≥ cutoff _= 163.

1. Baseline clinical characteristics of patients: In this study, we included 794 inpatients with CS-AKI. Of these, 77 patients had CKD before surgery, 1 patient had a malignant tumor, 125 patients died within 30 days after the occurrence of CS-AKI, and 27 cases did not have follow-up data for 90 days after surgery. Thus, after excluding these patients, 564 cases were finally included [414 males and 150 females, age: 57.55 ± 11.86 years]; 108 (19.1%) patients progressed to CKD within 90 days after the occurrence of CS-AKI. Patients with CS-AKI progressing to CKD were older and had a higher proportion of females; hypertension, diabetes, congestive heart failure, and coronary heart disease; preoperative use of angiotensin-converting enzyme inhibitors and angiotensin receptor blockers, calcium ion channel blockers, and beta-blockers; and preoperative low baseline eGFR, preoperative low hemoglobin level, and higher serum creatinine level at discharge (*P *< 0.05) than those without CS-AKI progressing to CKD, as shown in Table 1. No significant differences were observed between the two groups with respect to other indicators, including the proportion of preoperative cerebrovascular disease, peripheral vascular disease, COPD, diuretic use, CRRT, blood transfusion within 14 days after surgery, postoperative infection, atrial fibrillation; the AKI classification; the duration of mechanical ventilation; the reoperation rates for bleeding within 24 h after surgery, and other laboratory indicators (Table 1).
2. Risk factors for the progression to CKD in patients 90 days after the occurrence of CS-AKI: Clinical factors were used as predictors; the end point of the study was progression to CKD in patients 90 days after the occurrence of CS-AKI. The results of univariate logistic regression analysis showed that the progression of CS-AKI to CKD was associated with older age; female sex; combined hypertension, diabetes, coronary heart disease, congestive heart failure; high mean arterial pressure on admission; preoperative use of beta-blockers; preoperative low hemoglobin level, preoperative low baseline eGFR, high blood creatinine level at discharge; and other factors (Table 2). Incorporating the abovementioned factors into multivariate logistic regression analysis, the results showed that female sex, preoperative hypertension, coronary heart disease, congestive heart failure, preoperative low baseline eGFR, and higher serum creatinine level at discharge were independent risk factors associated with progression from CS-AKI to CKD (Table 3).
3. The efficacy of the clinical risk prediction model to predict the progression of CS-AKI to CKD: We selected the independent risk factors associated with the progression of CS-AKI to CKD in the multivariate regression analysis results (female sex, preoperative hypertension, coronary heart disease, congestive heart failure, preoperative low baseline eGFR, and higher serum creatinine level at discharge), including age, diabetes, hemoglobin level, mean arterial pressure, and other clinical factors to establish a comprehensive clinical risk prediction model. The regression model had good goodness of fit (Hosmer–Lemeshow test, chi-square level 5.693, *P *= 0.682); the area under the receiver operating characteristic curve for predicting progression from CS-AKI to CKD was 0.859 [95% *confidence interval* (*CI*): 0.823–0.896, *P *= 0.000], the sensitivity was 75.9%, the specificity was 82.2%, and the accuracy rate was 58.2% (shown in Figure 1).

**Table 1:** Comparison of baseline clinical characteristics of patients in CS-AKI-CKD group and CS-AKI-NCKD group.

Variable	CS-AKI-NCKD (*n* = 456)	CS-AKI-CKD (*n* = 108)	*P*
Age (y)	58 **(**49,65.75)	64 (56,68)	0**.**000
Female [*n* (%)]	109 **(**23.9)	41 **(**38.0)	0**.**003
Preoperative hypertension [*n* (%)]	234 **(**51.3)	73 **(**67.6)	0**.**002
Preoperative diabetes [*n* (%)]	48 **(**10.5)	22 **(**20.4)	0**.**005
Preoperative coronary heart disease [*n* (%)]	109 **(**23.9)	48 **(**44.4)	0**.**000
Preoperative congestive heart failure [*n* (%)]	154 **(**33.8)	51 **(**47.2)	0**.**009
Preoperative cerebrovascular disease [*n* (%)]	56 **(**12.3)	17 **(**15.7)	0**.**335
Preoperative peripheral vascular disease [*n* (%)]	10 **(**2.2)	2 **(**1.9)	0**.**825
Preoperative COPD [*n* (%)]	13 **(**2.9)	4 **(**3.7)	0**.**641
Preoperative use of ACEI/ARB [*n* (%)]	122 **(**26.8)	42 **(**38.9)	0**.**013
Preoperative use of beta-blockers [*n* (%)]	214 **(**46.9)	75 **(**69.4)	0**.**000
Preoperative use of calcium blockers [*n* (%)]	129 **(**28.3)	41 **(**38.0)	0**.**049
Preoperative use of diuretics [*n* (%)]	277 **(**60.7)	71 **(**65.7)	0**.**337
Intra-aortic balloon counterpulsation [*n* (%)]	54 **(**11.8)	12 **(**11.1)	0**.**832
Operation time(min)	420 **(**340,515)	405 **(**335,465)	0**.**054
Intraoperative blood loss(ml)	600 **(**350,900)	525 **(**312.5,800)	0**.**273
Body mass index (kg/m^2^)	25.4 (22.9,27.7)	25.1 **(**23.5,28.5)	0**.**453
Systolic blood pressure(mmHg)	128.5 **(**117,142)	133 **(**120,148)	0**.**033
Diastolic blood pressure(mmHg)	78 **(**69,87.8)	80 **(**70,88.8)	0**.**184
Mean arterial pressure(mmHg)	94 **(**86.7,105)	96.5 **(**90,107.7)	0**.**050
Preoperative ejection fraction (%)	58 **(**54,63)	57 **(**51,61)	0**.**034
Preoperative hemoglobin (g/L)	131 **(**118,144)	125.5 **(**113,137)	0**.**009
Preoperative albumin (g/L)	40.5 **(**37.8,42.9)	40.1 **(**37.3,42.9)	0**.**573
Preoperative blood urea nitrogen (mmol/L)	6.40 **(**5.10,8.08)	6.90 **(**5.63,8.35)	0**.**044
Preoperative serum creatinine (µmol/L)	77.8 **(**67.7,91.7)	85.0 **(**72.0,98.8)	0**.**001
Preoperative serum uric acid (mmol/L)	353 **(**289,428)	371.5 **(**280.5,451.3)	0**.**425
Baseline eGFR (ml · min^−1^ · 1.73 m^−2^)	93.1 **(**80.1,102.2)	78.6 **(**68.8,91.6)	0**.**000
AKI stage 1[*n* (%)]	117 **(**25.6)	21 **(**19.4)	0**.**306
Stage 2[*n* (%)]	196 **(**43)	54 **(**50)	
Stage 3[*n* (%)]	143 **(**31.4)	33 **(**30.6)	
Postoperative mechanical ventilation time(min)	3,750 **(**1,985, 6,220)	2,610 **(**1,820, 5,475)	0**.**075
Reoperation within 24 h after surgery [*n* (%)]	22 **(**4.8)	5 **(**4.6)	0**.**832
Postoperative CRRT [*n* (%)]	45 **(**9.9)	13 **(**12)	0**.**505
Postoperative atrial fibrillation [*n* (%)]	36 **(**7.9)	11 **(**10.2)	0**.**439
Blood transfusion within 14 days after surgery [*n* (%)]	323 **(**70.8)	78 **(**72.2)	0**.**775
Postoperative infection [*n* (%)]	421 **(**92.3)	103 **(**95.4)	0**.**268
Serum creatinine at discharge (µmol/L)	86.8**(**68.6,107.9)	137.5**(**110.0,166.5)	0**.**000

ACEI/ARB, angiotensin converting enzyme inhibitors and angiotensin receptor blockers.

**Table 2:** Influencing factors of progression to CKD in patients 90 days after CS-AKI (univariate logistic regression analysis).

Influencing factors	OR (95%CI)	*P*
Age(year)	1.045 (1.023,1.067)	0.000
Gender (female/male)	1.948 (1.249,3.038)	0.003
Preoperative hypertension(Y/N)	1.979 (1.271,3.081)	0.003
Preoperative diabetes(Y/N)	2.174 (1.247,3.790)	0.006
Preoperative congestive heart failure(Y/N)	1.755 (1.147,2.683)	0.009
Preoperative coronary heart disease(Y/N)	2.547 (1.646,3.940)	0.000
Preoperative use of ACEI/ARB(Y/N)	1.742 (1.123,2.702)	0.013
Preoperative use of beta-blockers(Y/N)	2.570 (1.641,4.026)	0.000
Mean arterial pressure on admission(mmHg)	1.014 (1.001,1.027)	0.034
Preoperative ejection fraction (%)	0.977 (0.955,0.999)	0.042
Preoperative hemoglobin(g/L)	0.988 (0.978,0.998)	0.021
Preoperative serum creatinine(µmol/L)	1.021 (1.008,1.033)	0.001
Baseline eGFR (ml · min^−1^ · 1.73 m^−2^)	0.949 (0.934,0.964)	0.000
Serum creatinine at discharge(µmol/L)	1.017 (1.013,1.022)	0.000

**Table 3:** Influencing factors of progression to CKD in patients 90 days after CS-AKI (multivariate logistic regression analysis).

Influencing factors	OR (95%CI)	*P*
Gender (female/male)	3.478 (1.844,6.559)	0.000
Preoperative hypertension (Y/N)	1.835 (1.046,3.220)	0.034
Preoperative coronary heart disease(Y/N)	1.779 (1.015,3.118)	0.044
Preoperative congestive heart failure(Y/N)	1.908 (1.124,3.239)	0.017
Baseline eGFR (ml · min^−1^ · 1.73 m^−2^)	0.956 (0.938,0.975)	0.000
Serum creatinine at discharge(µmol/L)	1.019 (1.014,1.024)	0.000

## Discussion

The results of our study showed that 19.1% of patients with CS-AKI developed CKD (eGFR < 60 ml · min^−1^ · 1.73 m^−2^) in 90 days after the occurrence of CS-AKI, which is consistent with the result of previous study (17). Patients with CS-AKI progressing to CKD were older and had a higher proportion of females; hypertension, diabetes, congestive heart failure, and coronary heart disease; preoperative use of angiotensin-converting enzyme inhibitors and angiotensin receptor blockers, calcium ion channel blockers, and beta-blockers; and preoperative low baseline eGFR, preoperative low hemoglobin level, and higher serum creatinine level at discharge than those without CS-AKI progressing to CKD. Multivariate logistic regression analysis showed that female sex, preoperative hypertension, coronary heart disease, congestive heart failure, preoperative low baseline eGFR, and higher serum creatinine level at discharge were independent risk factors for CS-AKI progressing to CKD (18). The clinical risk prediction model that included factors, such as older age; female sex; combined hypertension, diabetes, coronary heart disease, congestive heart failure; high mean arterial pressure on admission; preoperative use of beta-blockers; preoperative low hemoglobin level, preoperative low baseline eGFR, high blood creatinine level at discharge; and other factors, produced a moderate performance for predicting CS-AKI progression to CKD (area under receiver operator characteristic curve = 0.859, 95% *CI:* 0.823–0.896, *P *= 0.000), which is similar to the results of previous studies (19, 20).

Our study showed that the female sex is a risk factor for the progression of CS-AKI to CKD [*odds ratio* (*OR*)* *= 3.478], considering the possible reason that the ability of females to withstand major surgeries may be lower than that of males. This is similar to the conclusion that women have a significantly higher risk of progression to CKD after AKI than men from the Kidney Int (21) and JAMA (14), even incorporating it into the predictive scoring system as a reference variable for the progression to CKD after AKI (14).

Next, the results of our study showed that preoperative complications such as hypertension, coronary heart disease, and congestive heart failure are risk factors for the progression to CKD after CS-AKI. The surgical procedures used in our study population mainly included coronary artery bypass grafting, valve replacement or coronary artery bypass grafting combined with valve replacement, aortic arch replacement for Stanford type A aortic dissection, and a small number of other cardiac surgeries. It should be noted that coronary heart disease and coronary artery bypass grafting, congestive heart failure and valve replacement, and hypertension and aortic dissection (Stanford type A) aortic arch replacement have a more direct association. Cardiac surgery in patients with congenital heart disease is associated with an increased risk of AKI and CKD (21); some of these patients even have chronic heart failure. However, there are few reports in the literature regarding coronary heart disease and congestive heart failure as risk factors for CS-AKI progression to CKD. AKI is a common clinical complication associated with poor prognosis (22). Furthermore, a meta-analysis of AKI issues in type I cardiorenal syndrome found that nearly 1/4 of patients developed AKI, approximately 3% required renal replacement therapy, patients with acute heart failure had the highest incidence of AKI, and cardiac surgery patients had the greatest impact on mortality (23); however, there are no reports on long-term CKD. Our study is similar to previous ones in that the current literature or guidelines all believe that hypertension is a risk factor for the occurrence and development of CKD (24). Moreover, regarding preoperative comorbidities, patients with diabetes have a higher risk of AKI and CKD (25). The univariate logistic analysis results in our study showed that diabetes was a risk factor for CS-AKI to CKD; however, the results of the multivariate logistic analysis showed no statistical difference. The possible reason was that the patients included in the study might have both diabetes and coronary artery disease. There was a certain correlation between the two factors, but we still included diabetes as a risk factor in the final clinical risk prediction model; the prediction performance of this model was moderate.

In addition, our study identified preoperative low baseline eGFR and higher serum creatinine levels at discharge as risk factors for the development of CS-AKI to CKD, which is consistent with the previous reports in the literature (14, 17, 20). The baseline eGFR represents the patient's renal function reserve, and the serum creatinine level at discharge represents the patient's recovery after CS-AKI. The higher the serum creatinine level at discharge, the bigger the risk of developing CKD after 90 days after CS-AKI.

Some large-scale clinical retrospective studies have confirmed that AKI significantly increases the risk of subsequent CKD and end-stage renal disease in patients (4, 26). Therefore, KDIGO's 2012 AKI clinical practice guidelines pointed out that patients with AKI without CKD should also be regarded as a higher-risk group for CKD. It is recommended to evaluate the renal function of patients 90 days after the symptoms of AKI are relieved to determine whether there is a new development of CKD (15). Our study observed that 19.1% of patients developed CKD 90 days after the occurrence of CS-AKI, which was significantly higher than the 10.8% of the general population in China (27). Previous studies have similarly suggested that delayed AKI recognition is an independent risk factor for increased in-hospital mortality and that renal referral is an independent protective factor for AKI under-recognition and death (18). Our study further confirms that even if CS-AKI recovers, there are still patients who progress to CKD after 90 days, indicating that special attention should be paid to the evaluation of renal function and timely intervention after 90 days and increase kidney referrals to reduce the risk of conversion to CKD in the CS-AKI population.

Our study has the following advantages: First, the study population was relatively pure, including patients with CS-AKI; there are few clinical studies on the risk factors of CS-AKI progression to CKD, thus focusing on CS-AKI conversion to CKD has a special clinical guiding value. Second, this was a retrospective, observational cohort clinical study. The research endpoint of CS-AKI progression to CKD was formulated in accordance with international guidelines. The research team collected complete baseline data; preoperative, intraoperative, and postoperative data; and primary endpoint data to ensure the scientificity of the study and the reliability of the conclusions.

## Study limitations

There are some limitations of this study: (1) In a retrospective clinical study, there are no 90-day follow-up data for some patients, and some patients have incomplete preoperative detection indicators, such as cystatin C, hemoglobin A1c, and N-terminal-pro hormone BNP; however, these indicators were not included for univariate and multivariate analyses in this study and (2) Patients without AKI after cardiac surgery were not included for comparison to analyze the occurrence of CKD in this population.

## Conclusion

In conclusion, patients with CS-AKI are a higher risk group for new-onset CKD, and a comprehensive clinical risk prediction model consisting of sex, disease status, and preoperative eGFR can help identify high-risk patients with CS-AKI progressing to CKD; however, further perspective, multicenter, and long-term follow-up studies are needed to observe its long-term impact and predictive power on CKD.

## Data Availability

The raw data supporting the conclusions of this article will be made available by the authors, without undue reservation.

## References

[1] Li PK Burdmann EA Mehta RL. Acute kidney injury: global health alert. Kidney Int. (2013) 83:372–6. 10.1038/ki.2012.427 23302721

[2] Xu X Nie S Liu Z Chen C Xu G Zha Y Epidemiology and clinical correlates of aki in Chinese hospitalized adults. Clin J Am Soc Nephrol. (2015) 10:1510–8. 10.2215/CJN.02140215 26231194 PMC4559507

[3] Hsu CY. Linking the population epidemiology of acute renal failure, chronic kidney disease and end-stage renal disease. Curr Opin Nephrol Hypertens. (2007) 16:221–6. 10.1097/MNH.0b013e3280895ad9 17420665

[4] Coca SG Singanamala S Parikh CR. Chronic kidney disease after acute kidney injury: a systematic review and meta-analysis. Kidney Int. (2012) 81:442–8. 10.1038/ki.2011.379 22113526 PMC3788581

[5] Chawla LS Eggers PW Star RA Kimmel PL. Acute kidney injury and chronic kidney disease as interconnected syndromes. N Engl J Med. (2014) 371:58–66. 10.1056/NEJMra1214243 24988558 PMC9720902

[6] Heung M Steffick DE Zivin K Gillespie BW Banerjee T Hsu CY Acute kidney injury recovery pattern and subsequent risk of ckd: an analysis of veterans health administration data. Am J Kidney Dis. (2016) 67:742–52. 10.1053/j.ajkd.2015.10.019 26690912 PMC6837804

[7] Machado MN Nakazone MA Maia LN. Acute kidney injury based on kdigo (kidney disease improving global outcomes) criteria in patients with elevated baseline serum creatinine undergoing cardiac surgery. Rev Bras Cir Cardiovasc. (2014) 29:299–307. 10.5935/1678-9741.20140049 25372901 PMC4412317

[8] Ponte B Felipe C Muriel A Tenorio MT Liano F. Long-term functional evolution after an acute kidney injury: a 10-year study. Nephrol Dial Transplant. (2008) 23:3859–66. 10.1093/ndt/gfn398 18632586

[9] Ishani A Nelson D Clothier B Schult T Nugent S Greer N The magnitude of acute serum creatinine increase after cardiac surgery and the risk of chronic kidney disease, progression of kidney disease, and death. Arch Intern Med. (2011) 171:226–33. 10.1001/archinternmed.2010.514 21325112

[10] Schiffl H Lang SM Fischer R. Long-term outcomes of survivors of icu acute kidney injury requiring renal replacement therapy: a 10-year prospective cohort study. Clin Kidney J. (2012) 5:297–302. 10.1093/ckj/sfs070 25874084 PMC4393475

[11] Chawla LS Amdur RL Shaw AD Faselis C Palant CE Kimmel PL. Association between aki and long-term renal and cardiovascular outcomes in United States veterans. Clin J Am Soc Nephrol. (2014) 9:448–56. 10.2215/CJN.02440213 24311708 PMC3944753

[12] Sawhney S Marks A Fluck N Levin A McLernon D Prescott G Post-discharge kidney function is associated with subsequent ten-year renal progression risk among survivors of acute kidney injury. Kidney Int. (2017) 92:440–52. 10.1016/j.kint.2017.02.019 28416224 PMC5524434

[13] Goldstein SL Jaber BL Faubel S Chawla LS. Aki transition of care: a potential opportunity to detect and prevent ckd. Clin J Am Soc Nephrol. (2013) 8:476–83. 10.2215/CJN.12101112 23471414

[14] James MT Pannu N Hemmelgarn BR Austin PC Tan Z McArthur E Derivation and external validation of prediction models for advanced chronic kidney disease following acute kidney injury. JAMA. (2017) 318:1787–97. 10.1001/jama.2017.16326 29136443 PMC5820711

[15] Kellum JA Lameire N Aspelin P Barsoum RS Burdmann EA Goldstein SL Kidney disease: improving global outcomes (kdigo) acute kidney injury work group. Kdigo clinical practice guideline for acute kidney injury. (2012).

[16] Inker LA Eneanya ND Coresh J Tighiouart H Wang D Sang Y New creatinine- and cystatin c–based equations to estimate gfr without race. N Engl J Med. (2021) 385:1737–49. 10.1056/NEJMoa2102953 34554658 PMC8822996

[17] Palomba H Castro I Yu L Burdmann EA. The duration of acute kidney injury after cardiac surgery increases the risk of long-term chronic kidney disease. J Nephrol. (2017) 30:567–72. 10.1007/s40620-016-0351-0 27704389

[18] Yang L Xing G Wang L Wu Y Li S Xu G Acute kidney injury in China: a cross-sectional survey. Lancet. (2015) 386:1465–71. 10.1016/S0140-6736(15)00344-X 26466051

[19] Chawla LS Amdur RL Amodeo S Kimmel PL Palant CE. The severity of acute kidney injury predicts progression to chronic kidney disease. Kidney Int. (2011) 79:1361–9. 10.1038/ki.2011.42 21430640 PMC3257034

[20] Luo W Li J Jiang J Yang X. Characteristics and risk factors of acute kidney injury progressed to chronic kidney disease: a prospective, observational cohort study. Chin J Nephrol. (2020) 36:625–30. 10.3760/cma.j.cn441217-20200528-00112

[21] Madsen NL Goldstein SL Froslev T Christiansen CF Olsen M. Cardiac surgery in patients with congenital heart disease is associated with acute kidney injury and the risk of chronic kidney disease. Kidney Int. (2017) 92:751–6. 10.1016/j.kint.2017.02.021 28412020

[22] Marenzi G Assanelli E Campodonico J De Metrio M Lauri G Marana I Acute kidney injury in st-segment elevation acute myocardial infarction complicated by cardiogenic shock at admission. Crit Care Med. (2010) 38:438–44. 10.1097/CCM.0b013e3181b9eb3b 19789449

[23] Vandenberghe W Gevaert S Kellum JA Bagshaw SM Peperstraete H Herck I Acute kidney injury in cardiorenal syndrome type 1 patients: a systematic review and meta-analysis. Cardiorenal Med. (2016) 6:116–28. 10.1159/000442300 26989397 PMC4789882

[24] Allison SJ. Acute kidney injury: ckd, aki and outcomes in acute severe hypertension. Nat Rev Nephrol. (2010) 6:384. 10.1038/nrneph.2010.75 20597157

[25] Hapca S Siddiqui MK Kwan R Lim M Matthew S Doney A The relationship between aki and ckd in patients with type 2 diabetes: an observational cohort study. J Am Soc Nephrol. (2021) 32:138–50. 10.1681/ASN.2020030323 32948670 PMC7894655

[26] Ishani A Xue JL Himmelfarb J Eggers PW Kimmel PL Molitoris BA Acute kidney injury increases risk of esrd among elderly. J Am Soc Nephrol. (2009) 20:223–8. 10.1681/ASN.2007080837 19020007 PMC2615732

[27] Zhang L Wang F Wang L Wang W Liu B Liu J Prevalence of chronic kidney disease in China: a cross-sectional survey. Lancet. (2012) 379:815–22. 10.1016/S0140-6736(12)60033-6 22386035

